# The Influence of Ripeness on the Phenolic Content, Antioxidant and Antimicrobial Activities of Pumpkins (*Cucurbita moschata* Duchesne)

**DOI:** 10.3390/molecules26123623

**Published:** 2021-06-13

**Authors:** Meriem Mokhtar, Sarah Bouamar, Arianna Di Lorenzo, Caterina Temporini, Maria Daglia, Ali Riazi

**Affiliations:** 1 Laboratory of Beneficial Microorganisms, Functional Food and Health (LMBAFS), University of Abdelhamid Ibn Badis, Mostaganem 27000, Algeria; sarah.bouamar@univ-mosta.dz (S.B.); ali.riazi@univ-mosta.dz (A.R.); 2 Department of Drug Sciences, Medicinal Chemistry and Pharmaceutical Technology, University of Pavia, Via Taramelli 12, 27100 Pavia, Italy; arianna.dilorenzo01@universitadipavia.it; 3 Department of Drug Sciences and Italian Biocatalysis Center, University of Pavia, Via Taramelli, 12, 27100 Pavia, Italy; caterina.temporini@unipv.it; 4 Department of Pharmacy, University of Naples “Federico II”, 80131 Naples, Italy; maria.daglia@unina.it; 5 International Research Center for Food Nutrition and Safety, Jiangsu University, Zhenjiang 212013, China

**Keywords:** *Cucurbita moschata*, polyphenols, ripening, antioxidant, antimicrobial

## Abstract

*Cucurbita moschata* Duchesne (*Cucurbitaceae*) is a plant food highly appreciated for the content of nutrients and bioactive compounds, including polyphenols and carotenoids, which contribute to its antioxidant and antimicrobial capacities. The purpose of this study was to identify phenolic acids and flavonoids of *Cucurbita moschata* Duchesne using high-performance liquid chromatography–diode array detection–electrospray ionization tandem mass spectrometry (HPLC–DAD–ESI-MS) at different ripening stages (young, mature, ripened) and determine its antioxidant and antimicrobial activities. According to the results, phenolic acids and flavonoids were dependent on the maturity stage. The mature fruits contain the highest total phenolic and flavonoids contents (97.4 mg GAE. 100 g^−1^ and 28.6 mg QE. 100 g^−1^).A total of 33 compounds were identified. Syringic acid was the most abundant compound (37%), followed by cinnamic acid (12%) and protocatechuic acid (11%). Polyphenol extract of the mature fruits showed the highest antioxidant activity when measured by DPPH (0.065 μmol TE/g) and ABTS (0.074 μmol TE/g) assays. In the antimicrobial assay, the second stage of ripening had the highest antibacterial activity. *Staphylococcus aureus* was the most sensitive strain with an inhibition zone of 12 mm and a MIC of 0.75 mg L^−1^. The lowest inhibition zone was obtained with *Salmonella typhimurium* (5 mm), and the MIC value was 10 mg L^−1^.

## 1. Introduction

According to several epidemiological studies, the consumption of fruits and vegetables exert protective effects against several risk factors of chronic diseases because of their content of micronutrients, dietary fiber, and phytochemicals [1,2,3,4,5]. In addition, the interest in plant foods is also due to their antioxidant and antimicrobial properties that make plant extracts able to act against both lipid peroxidation and foodborne bacteria. Considering the growing demand to replace synthetic preservatives such as methylparabens with natural substances, research on preservatives is of great interest to the food and cosmetic industry [6,7,8,9].

Pumpkin, *Cucurbita moschata* Duchesne (*Cucurbitaceae*), is an essential source of many antioxidant nutrients such as polyphenols and carotenoids and is cultivated in warm areas all over the world [10,11]. There are several culinary uses of squashes; they can be used as a vegetable or as an ingredient in food preparations [12,13]. In addition, pumpkin fruits are rich in many essential compounds for the human body, such as eight amino acids, vitamins (A, B, C), various minerals, carotene, and trace elements (phosphorus, potassium, calcium, magnesium, zinc, and silicon) [14].

The chemical composition and biological properties of different parts of *C. moschata* have been examined in many investigations confirming that pumpkins have a wide range of bioactivities, such as anti-diabetes, anti-inflammation, hepatoprotective, anticancer, and anti-obesity properties [15,16,17,18,19,20,21,22]. These properties are generally attributedto the content of phenolic acids [23].

Ten days after pollination, the fruit is characterized by a small volume and has reached the young fruit stage. Twenty days after flowering, a rapid increase of size and accumulation of metabolites occurs, and the pumpkin fruit enters the expanding stage. Ten days later, the fruit enters a premature stage reaching a maximum volume. Forty days after flowering, the fruit is considered to be fully mature. Once the fruit comes to its full size (50 days), the ripening process is initiated, and significant biochemical changes occur in the maturing fruit, which is associated with further dramatic changes in color, sweetness, and fruit texture [24].

Several intrinsic and extrinsic factors can influence polyphenol quality and quantity, including plant genetics, growing conditions (temperature, light, water, soil type, mineral nutrients, oxygen), and physiological maturity [25,26,27]. Some biochemical, physiological, and structural modifications occur during ripening and affect the quality of the fruits.The fruit undergoes some changes in gene expression level, resulting in some desirable variations such as texture and firmness, sugar accumulation, organic acid reduction, pigment changes that lead to the development of color, and volatile compounds responsible for flavor and aroma [28]. As reported in the literature, the phytochemistry variation depends on the phytochemical’s biosynthesis during plant growth and their changes during physiological maturity [29].

The knowledge of the chemical composition and functional properties during ripening is essential to characterize the perfect harvesting time and maximize the antimicrobial and antioxidant properties to exploit a plant extract as a food preservative. Considering that no previous study has been conducted on identifying individual phenolic compounds of *C. moschata* during maturation, this work aimed to study the phenolic profile changes during ripening, besides the development of antioxidant and antimicrobial activities.

## 2. Results

### 2.1. Total Polyphenols and Flavonoids

Fruits and vegetables containing polyphenols are considered significant dietary sources of health-promoting components. The total phenolic and flavonoid content in pumpkins at the three stages of ripeness were estimated using the Folin–Ciocalteu and aluminum chloride colorimetric methods.

Our results showed that pumpkin total phenolic and flavonoid contents are dependent on the fruit maturity stage (Table 1). The unripe stage contains a good quantity of total polyphenols (77.5 mg GAE. 100 g^−1^ FW) and flavonoids (23.4 mg QE. 100 g^−1^ FW). These amounts increased with the fruit ripeness and were 26 and 22% higher in the second stage than unripe fruits (97.4 mg GAE. 100 g^−1^ and 28.6 mg QE. 100 g^−1^).

At the end of fruit ripeness, the levels of both polyphenols and flavonoids decrease (55.6 mg GAE. 100 g^−1^ and 19.6 mg QE. 100 g^−1^). These findings suggest that the phenolic content increase during the first stages of maturity, but these levels start to drop off with the ripeness of the fruit. The decrease in total phenolic content during fruit maturity was referred to as the oxidation of polyphenols by polyphenol-oxidase [30,31].

Oloyede et al. [32] found that mature pumpkin fruits have a 71% higher polyphenol content than young pumpkin fruits. Proanthocyanidin, anthocyanin, and flavonoid concentrations were also more important in mature fruits (17%, 14%, and 4.5%, respectively). The phenolic contents of their immature and mature samples were 10.3 and 33.5 mg/100 g. For the flavonoids, 5.4 and 6.0 mg/100 g were calculated. The total content of polyphenols and flavonoids obtained in our samples was more important but lower than that reported by Zdunić et al. [33] (90.59 mg/100 g).

A series of complex biochemical reactions occur during fruit ripening that leads to the production of phenolic compounds, carotenoids, and volatile compounds [34]. Several factors are responsible for the qualitative and quantitative differences of the above-listed phytochemicals, such as sunlight, soils, season, cultivation area, variety of fruit, and maturity stages [35].

### 2.2. Analysis of Polyphenols by HPLC–PDA–ESI-MS

**Figure 1:**
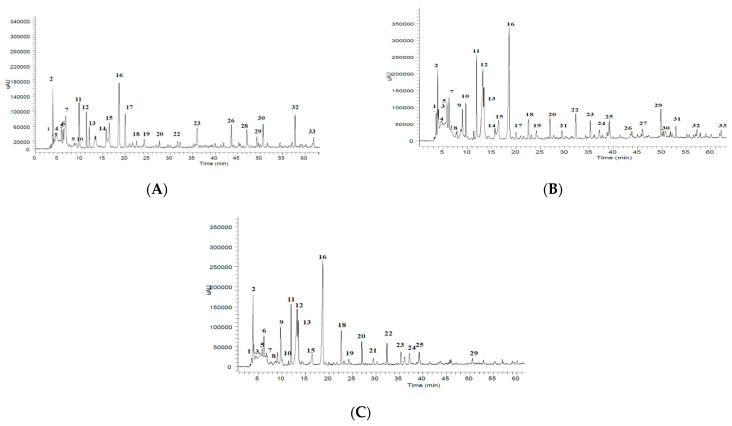
Identification of *C. moschata* polyphenols in (**A**) young, (**B**) mature, and (**C**) ripened fruits.

In an attempt to characterize polyphenols in the three stages of maturity of pumpkin fruits, other polar compounds were also identified. The phenolic profile at the three stages of ripeness of pumpkins is presented in Figure 1. A total of 33 compounds were identified, and among them, phenolic acids are the main components (Table 2). Phenolic acids represent approximately 30% of the dietary polyphenols and are present in both free and bound forms in plants [36].

These results are consistent with previous studies in different varieties of pumpkins [20,37,38]. Although several works have reported the presence of chlorogenic acid [11,23,39], it was not detected in our sample. Some flavonoids were also identified in pumpkin extracts, mainly quercetin glucoside. In addition, another phenolic compound belonging to the lignan class was identified (Lariciresinol-sesquilignan). This compound has already been reported in other researches on pumpkins [40,41].

For the other polar compounds, five amino acids were detected (valine, threonine, tyrosine, phenylalanine, and aspartic acid), two organic acids (tartaric acid and feruloyl malate), and one glycosylamine (adenosine). These polar compounds were also described in a previous work conducted by Iswaldi et al. [42] in *Cucurbita pepo*.

Quantitative determination of pumpkin polyphenols was performed by interpolation of the calibration curves, and the results (μg/g ± SD) are reported in Table 3. Syringic acid was the most abundant compound representing 37% of the phenolic compounds of pumpkins, followed by cinnamic acid (11.93%), protocatechuic acid (11.30), *p*-comaroylhexoside (4.71%), quercetin glucoside (4.53%), and vanillin (4.29%).

Fruit or other plant tissue maturation involves a series of complex reactions which lead to changes in the plant’s phytochemistry. Two distinct phenomena of phenolic content change were observed during ripeness: a steady decrease [43,44] or an increase at the end of ripeness [45,46].

According to the results, some phenolic acids such as (caffeic acid, caffeic acid derivative, protocatechuic acid, cinnamic acid, syringic acid, coumaric acid, dihydroferulic acid, *p*-comaroylhexoside, and vanillin) and flavonoid compounds (quercetin glucoside, luteolin 7-*O*-glucuronide, luteolin-7-*O*-rutinoside, and (−)-gallocatechin gallate) significantly increase from young to mature stages. However, those levels are reduced in the ripened fruits (*p* < 0.05).

The concentrations of dihydroferulic acid derivative and catechin continue to increase from young to ripened fruits (*p* < 0.05); they were 48% and 30% higher than the levels obtained in the mature fruits.Ndri et al. [47] studied polyphenols at different ripeness stages in Gnagnan (*Solanum indicum* L.) berries. Their results suggest that as maturity progresses, the amount of phenolic acids increases. These results are, in general, similar to those found in the present study.

Previous research carried out by Gündoğdu et al. [48] has reported similar findings. These authors studied the development of phenolic compounds during three maturity stages in apricots (*Rosaceae*). They noticed that the mid-ripe stage was the richest in both phenolic acids and flavonoids.

On the other hand, the amounts of sinapic acid, ferulic acid, caftaric acid, protocatechuic acid derivate, (−)-epicatechin gallate, and quercetin-hexosidewere the highest in the first stage and decreased with maturity (*p* < 0.05).

### 2.3. Antioxidant Activity

Oxidation is considered a major cause of food and food product deterioration. Different assays describing the capability of redox molecules to scavenge free radicals to measure the antioxidant capacity of food and biological samples can be found in the literature [49,50,51].

The mature stage showed the highest antioxidant activity compared to the other stages when measured by DPPH (0.065 ± 0.010 μmol TE/g), and ABTS (0.074 ± 0.021 μmol TE/g) assays (Table 4). By contrast, the pumpkin ripened stage exerted the lowest antioxidant activity with a loss of 37% and 27% in the DPPH and ABTS tests. Thus, the antioxidant activity reduction during pumpkin fruit maturation may be associated with the relative decrease in the content of various polyphenol compounds [52,53].

A positive correlation is observed between the antioxidant capacity of pumpkin polyphenols at different stages of fruit maturation in relation to total phenolic and flavonoid contents. Both DPPH and ABTS are strongly correlated with total polyphenols (R^2^ = 0.996 and R^2^ = 0.984) and flavonoids (R^2^ = 0.997 andR^2^ = 0.945) respectively.

Due to their capacity to donate hydrogen atoms to free radicals, phenolic and flavonoid molecules are significant antioxidant components that deactivate free radicals. They also have excellent structural properties for scavenging free radicals. Several studies have reporteda linear correlation between total phenolic and flavonoid contents and antioxidant activity. By analyzing the correlation coefficients (R-values), we can suggest that phenolic and flavonoid compounds are responsible for the antioxidant activity of the pumpkin extracts [54].

Syringic acid was identified as the major phenolic compound pumpkins with 37%. It is a natural phenolic acid found in many fruits and vegetables such as pumpkins, olives, grapes, rice, wheat, oats, maize, sorghum, sugar cane, and honey [55,56]. Many studies reported its biological activities, including antioxidant properties, anticancer, anti-inflammation, anti-diabetic, and antimicrobial [57,58,59,60]. In addition, several studies indicated that syringic acid exhibits an excellent radical scavenging activity against β-carotene and 2,2-diphenyl-1-picrylhydrazyl (DPPH) [55].

Cinnamic acid, among the most abundant polyphenols identified in our sample, is a natural aromatic carboxylic acid found in several plants such as Chinese cinnamon, ginseng, fruits, vegetables, whole grains, and honey [61,62]. According to the literature, cinnamic acid exhibits antioxidant, antimicrobial, anticancer, neuroprotective, anti-inflammatory, and anti-diabetic properties [63,64,65,66,67]. The antioxidant properties of cinnamic acid are attributed to its ability to end radical chain reactions by donating electrons that react with radicals forming stable products [62,68].

Protocatechuic acid (3,4-dihydroxybenzoic acid), also found in pumpkins, is a catechol-type phenolic acid that naturally exists in many fruits and vegetables and is known to have antibacterial, anti-mutagenic, anti-inflammatory, and anti-hyperglycemic properties. It has also been recognized as an effective antioxidant agent against oxidative stress, preventing several pathologies [69,70].

### 2.4. Antimicrobial Activity

Food deterioration during storage is a significant problem and concern for the food industry. Spoilage microorganisms are responsible for product degradation and short shelf-life. Over the last decade, the food and cosmetic industries have tried to reduce the use of chemical preservatives in response to the increasing demand for natural compounds with antimicrobial activity [71]. The obtained results of the filter disc are shown in Table 5.

According to the results, the second stage of maturation had a higher antibacterial activity, followed by the first and third stages. This could be explained by the phenolic content of each stage as the second stage had the highest total phenolics and flavonoids. The response of the strains to the same sample also varies.

It was also observed that gram-negative bacteria were more resistant to pumpkin polyphenols than gram-positive strains. In gram-positive bacteria, there is a thick layer of peptidoglycan that protects a single bilayer. On the other hand, the cell envelope of gram-negative bacteria consists of a thin layer of peptidoglycan surroundedbyanouter lipopolysaccharidemembrane [72]. Gram-negative bacteria have an extra layer playing an essential role in protecting from the hostile environment by excluding toxic molecules without compromising the exchange of material needed for sustaining life [73].

*Staphylococcus aureus* was the most sensitive strain to *Cucurbita* phenolic extract with an inhibition zone of 12 mm and a MIC of 0.75 mg L^−1^. The lowest inhibition zones were obtained with *Salmonella typhimurium* (5 mm). This resistance was also observed with the MIC value (10 mg L^−1^).

The major polyphenols identified in pumpkins belong to the phenolic acid class. Phenolic acids appeared to exhibit more significant antimicrobial activity than flavonoids. Thus, microbial transformations of some flavonoids could lead to more powerful compounds with a more significant antimicrobial effect (phenolic acids) that selectively affect intestinal bacteria [74,75,76,77,78]. Gallic acid, caffeic acid, and ferulic acid had better antimicrobial activity against Gram-positive and Gram-negative bacteria than gentamicin and streptomycin [79].

Polyphenolic compounds are excellent natural antimicrobial agents. Although the exact mechanism responsible for the antibacterial activity is still not well understood, several possible action mechanisms were described. For example, polyphenols might induce cell wall damage resulting in leakage of intracellular components such as ions, ATP, nucleic acids, and amino acids. Moreover, cell damage may also be related to nutrient uptake, inhibiting DNA synthesis by suppressing gyrase activity, influencing protein biosynthesis, blocking ATP synthesis, reducing the pH level inside the bacterial cell, and affecting biofilm formation [80,81,82,83].

Both beneficial strains *Bifidobacterium animalis* sbsp *lactis* (Bb12) and *Lactobacillus rhamnosus* LbRE-LSAS were resistant to polyphenol extracts. Prior studies have demonstrated that phenolic compounds have a selective effect, as they inhibit the growth of pathogens and stimulate commensal bacteria and probiotics, including *Lactobacillus* and *Bifidobacterium* species. Polyphenols tend to provide health benefits by exerting prebiotic-like effects and modulating the gut microbiota [84,85]. The potential prebiotic effects of food polyphenols are associated with the ability of intestinal microbiota to convert phenolics into their metabolites, which in turn contribute to modulate intestinal microbiota [86,87].

The antimicrobial activity of plant extracts has been demonstrated mainly in vitro. Nevertheless, some aspects are crucial for its use in foods. The activity of these compounds in bothinvitroand on the foodstuffs once applied tests are different; this could be explained by the presence of fat, carbohydrate, protein, salt, and pH, which may influence the effectiveness of these agents in foods [88,89,90,91]. Moreover, the availability of nutrients in food preparations compared with the culture media may enable the fast repair of damaged cells by the respective bacteria. Besides that, high levels of fat and/or protein in the foodstuffs might protect bacteria from the action of these natural extracts [89]. Storage temperature may also influence the effectiveness of antimicrobials as the diffusibility of compounds is related to the temperature [92,93]. Finally, and most importantly, we have to consider the effects of the extract on the sensory characteristics of the food products and make sure that the use of these natural preservatives cannot alter the organoleptic properties of food [91,93].

## 3. Material and Methods

### 3.1. Plant Material

The fruits of pumpkin were collected at three different development stages (i.e., young, mature, and ripened) from the region of Mostaganem (Algeria) during May 2018. Three fruit samples from each stage of development were selected based on their morphological attributes such as size, weight, and color. Fruits were washed, air-dried and the physical parameters were recorded.

### 3.2. Polyphenol Extraction

The extraction was carried out using the method of Mokhtar et al. [27]. A sample of 200 g of each stage had undergone two successive extractions, the first one using methanol as a solvent and the second one with ethyl acetate (0.05% *v*/*v* hydrochloric acid/solvent (10:90)) under sonication for 30min. Both extracts were combined, filtered, and evaporated until dry. The entire process is conducted in darkness and repeated in triplicate.

### 3.3. Phytochemical Investigations

#### 3.3.1. Determination of Total Phenolics

The Folin–Ciocalteau reagent method is used to determine total polyphenols [94]. A sample of 100 µL of each extract is added to 4.9 mL of distilled water in a 10 mL volumetric flask. Folin–Ciocalteau’s phenol reagent (0.5 mL; 2 N) is mixed with the solution. Three minutes later, 1 mL of a saturated sodium carbonate solution (35% *w*/*v*) is added into the mixture, following by topping it up to 10 mL with water. After 30 min of incubation, the absorbance is measured at 725 nm using a spectrophotometer. The content is expressed as mg of gallic acid equivalents/100 g of fruit.

#### 3.3.2. Determination of Total Flavonoids

Total flavonoids of pumpkins were determined according to the method of Pothirat et al. [95]. A sample of 500 µL of the extracts collected at three stages of ripeness is mixed with the same amount of 2% aluminum chloride solution. After 10 min of reaction, the absorbance is measured at 415 nm against a blank that contains the sample without aluminum chloride. The results are expressed as mg of quercetin equivalents/100 g of fruit.

#### 3.3.3. Identification of Phenolic Compounds by HPLC-DAD-ESI-MS

The chromatographic analyses were performed using a Thermo Finnigan Surveyor Plus HPLC apparatus consisting of a quaternary pump, a Surveyor UV-Vis photodiode array (PDA) detector, and LCQ Advantage max ion trap mass spectrometer (Thermo Fisher Scientific, Waltham, MA, USA), coupled through an electrospray ionization (ESI) source.

The separation was conducted on a Gemini C18 110 Å (150 × 2 mm, 5 μm). Water (A) and methanol (B) were used as mobile phases; both were acidified with 0.075% of formic acid. The gradient elution was as follows: 0–5 min: 2% of B, 5–120 min: 2–100% of B. The flow rate was 1 mL/min, and the injection volume was 2 μL [27]. Spectral data were collected in the range of 200–800 nm for all peaks, and the chromatograms were recorded at λ = 280 nm. HPLC-ESI-MS/MS data were obtainedinbothpositive and negative ionization modes. The external standard method was used to quantify each compound, and the results were expressed as µg g^−1^ fresh weight ± SD.

### 3.4. Antioxidant Activity Measurement

A wide range of spectrophotometric assays has been used in recent years to measure the antioxidant capacity of foods and biological samples. Among the most frequently used methods for determining antioxidant capacity are the ABTS and DPPH assays [50,51].

#### 3.4.1. DPPH Test

The free radical-scavenging capacity of *Cucurbita* polyphenols is tested as bleaching of the stable radical DPPH. The DPPH assay is done according to the method of Siracusa et al. [96]. DPPH• has a maximum absorbance at 517 nm; its deep violet color becomes colorless when converted into DPPH-H. A volume of 37.5 µL of the tested samples is added to 1.5 mL of DPPH (0.1 mmol). After an incubation time of 20 min, the absorbance is measured at 517 nm.Trolox is used as the standard, and the tested sample is replaced by methanol for the control. The concentrations are reported as μmol TE/g (TE = Trolox Equivalent/g of extract).

#### 3.4.2. Trolox Equivalent Antioxidant Capacity (TEAC)

The ABTS solution [2,2-azinobis-(3-ethylbenzothiazoline-6-sulfonic acid)] is prepared 12 to 14 hours earlier and is kept in darkness by mixing 1.7 mmol L^−1^ of ABTS to 4.3 mmol.L^−1^ of potassium peroxydisulfate in water in the ratio of 5:1. The concentrated ABTS^•^ solution is diluted with methanol to reach a final absorbance of 0.70±0.02 at 734 nm. Next, a stock solution of Trolox is prepared in methanol at different concentrations ranging between 0 and 250 mmol. A volume of 0.1 mL (tested sample/ standard/methanol for the control) is added to 2 mL of ABTS• solution, and the absorbance is measured at 734 nm after 6min against a blank. The TEAC of different stages of *Cucurbita* polyphenols is calculated by relating this decrease in absorbance to that of a Trolox solution on a molar basis [97].

### 3.5. Antimicrobial Activity

#### 3.5.1. Microbial Strains

Staphylococcus aureus ATCC 6538,Listeria monocytogenes ATCC 7644, Escherichia coli ATCC 10536; Salmonella typhimurium ATCC 13311,Enterococcus hirae ATCC 10541, Proteus mirabilis ATCC 13315, Bacillus subtilis ATCC 6633, Shigella dysenteriae CECT 457, Pseudomonas aeruginosa ATCC 27853, Bifidobacterium animalis sbsp lactis (Bb12), and Lactobacillus rhamnosus LbRE-LSAS were obtained from the LMBAF laboratory (University of Mostaganem, Mostaganem, Algeria).

#### 3.5.2. Antimicrobial Screening

The antimicrobial activity of the pumpkin polyphenols is determined according to the agar disc diffusion method. Bacterial inocula are prepared 16h earlier in Muller–Hinton broth (MHB, Oxoid) at 37 °C, and adjusted to 10^8^ CFU/mL prior to use. A volume of 20 µL of each stage is deposited on sterile filter paper disks (6mm). Another disk containing DMSO is also placed on the plate as a negative control. To ensure compound diffusion, the plates are left for 30 min at room temperature and then incubated at 37 °C. After 24 h, the inhibition zone diameter is measured [98].

The pumpkin stage with the best inhibition zones is selected to determine the minimum inhibitory concentration (MIC) by the broth microdilution method [98]. Bacterial suspension of 100 μL is added to the wells of a sterile 96-well microtiter plate already containing 100 μL of the diluted tested samples and incubated for 24 h at 37 °C. The final concentration of the inoculum was approximately 5 × 10^5^ CFU/mL.

### 3.6. Statistical Analysis

The statistical analysis is performed using Statbox pro (6.40). Each experiment was repeated three times, and the results are expressed as means ± SD.Differences between the means are evaluated using one-way ANOVA, and is considered significant at *p* ≤ 0.05.

## 4. Conclusions

Pumpkin is a valuable source of bioactive compounds responsible for several biological activities. The current research is a preliminary study that focused on the development of the phenolic profile of *Cucurbita moschata* during different maturation stages and their antioxidant and antimicrobial activities. Among the three stages, high levels of polyphenols were determined in the mature fruits. After the analysis with HPLC-DAD-ESI-MS, 33 compounds were identified, and syringic acid was the most important. Polyphenol extract of the mature fruits showed the highest antioxidant and antimicrobial activities, which can be attributed to high levels of phenolic acids and flavonoids. The obtained results in the present work contribute to highlight that pumpkins may be considered useful as a food preservative. However, further studies should be conducted to study the effectiveness of pumpkin polyphenols as a natural preservative in a food model.

## Figures and Tables

**Table 1:** Total polyphenols and flavonoids of *C. moschata* at three stages of ripeness.

Stages of Ripeness	Total Polyphenols (mg GAE. 100 g^−1^ FW)	Total Flavonoids (mg QE. 100 g^−1^ FW)
Young	77.50 ± 1.01	23.47 ± 0.90
Mature	97.43 ± 2.20	28.66 ± 0.33
Ripened	55.60 ± 3.60	19.60 ± 1.25

**Table 2:** Identification of *C. moschata* polyphenols by HPLC-DAD-ESI-MS.

N	RT	Molecular Formula	[M–H]^−^	MS^2^	Compounds
1	3.60	C_9_H_8_O_4_	215	215 179100 135	Caffeic acid derivative
2	3.83	C_15_H_18_O_8_	325	163119	*p*-comaroylhexoside
3	4.01	C_5_H_11_NO_2_	116	1166983	Valine
4	4.62	C_4_H_9_NO_3_	118	118101 72	threonine
5	5.78	C_8_H_8_O_3_	151	151	vanillin
6	6.18	C_11_H_14_O_4_	209	209	Sinapyl alcohol
7	6.75	C_11_H_12_O_5_	223	180 195	Sinapic acid
8	9.00	C_14_H_14_O_8_	505	179 132	Feruloyl malate
9	9.72	C_4_H_7_NO_4_	132	132 86	Aspartic acid
10	11.32	C_9_H_11_NO_3_	180	180	Tyrosine
11	11.99	C_7_H_6_O_4_	153	111 109	Protocatechuic acid
12	13.22	C_9_H_8_O_2_	147		cinnamic acid
13	13,44	C_4_H_6_O_6_	149		Tartaric acid
14	15.73	C_7_H_6_O_4_	157	109	Protocatechuic acid derivate
15	16.46	C_10_H_13_N_5_O_4_	266	136	adenosine
16	18.63	C_9_H_10_O_5_	197	166 120	syringic acid
17	19.97	C_9_H_11_NO_2_	164	120	Phenylalanine
18	22.59	C_10_H_12_O_4_	195	181	Dihydroferulic acid derivative
19	24.23	C_9_H_8_O_3_	163		coumaric acid
20	27.62	C_9_H_8_O_4_	179		caffeic acid
21	29.46	C_10_H_12_O_4_	195	178	Dihydroferulic acid
22	32.32	C_9_H_10_O_4_	181		Syringaldehyde
23	35.99	C_10_H_10_O_4_	193		Ferulic acid
24	37.14	C_15_H_14_O_6_	289		Catechin
25	39.22	C_13_H_8_O_6_	259	215187 231	1,3,5,6-tetrahydroxyxanthone
26	43.63	C_13_H_12_O_9_	311		Caftaric acid
27	46.06	C_22_H_18_O_11_	457		Gallocatechin gallate
28	47.07	C_30_H_36_O_10_	555		Lariciresinol-sesquilignan
29	49.83	C_21_H_20_O_12_	463	301	Quercetin glucoside
30	50.69	C_22_H_18_O_10_	441	539	Epicatechin gallate
31	52.92	C_21_H_18_O_12_	461		Luteolin-7-*O*-glucuronide
32	57.79	C_21_H_19_O_12_	455	301	Quercetin-hexoside
33	62.21	C_21_H_19_O_11_	593	285	Luteolin-7-*O*-rutinoside

**Table 3:** Quantification of *C. moschata* polyphenols in young, mature and ripened fruits (μg/g).

N	Compounds	Young	Mature	Ripened
1	Caffeic acid derivative	23.80 ± 1.41	102.58 ± 8.69	33.64 ± 2.60
2	*p*-comaroylhexoside	75.32 ± 2.17	171.14 ± 10.57	92.89 ± 5.84
3	Vanillin	10.74 ± 1.85	156.10 ± 7.69	43.99 ± 4.41
4	Sinapicacid	136.92 ± 8.21	27.16 ± 1.58	16.27 ± 1.41
5	Protocatechuicacid	96.90 ± 3.47	410.31 ± 13.99	229.46 ± 14.75
6	Cinnamic acid	43.04 ± 2.84	433.48 ± 19.27	316.46 ± 13.86
7	Protocatechuic acid derivate	228.79 ± 17.15	85.92 ± 4.31	-
8	Syringic acid	604.24 ± 18.55	1340.59 ± 28.12	998.55 ± 13.54
9	Dihydroferulic acid derivative	37.44 ± 2.38	105.66 ± 7.54	156.87 ± 8.78
10	Coumaric acid	33.33 ± 2.24	52.56 ± 4.87	30.56 ± 2.86
11	Caffeic acid	29.79 ± 1.79	106.80 ± 7.71	85.67 ± 5.88
12	Dihydroferulic acid	-	41.06 ± 3.15	29.64 ± 2.45
13	Ferulic acid	112.42 ± 7.98	85.24 ± 4.55	57.51 ± 3.84
14	Catechin	-	45.95 ± 2.21	59.79 ± 4.86
15	Caftaric acid	106.49 ± 9.15	43.35 ± 3.95	-
16	(−)-Gallocatechin gallate	-	41.21 ± 2.87	-
17	Quercetin glucoside	77.38 ± 5.66	164.66 ± 11.74	46.98 ± 3.27
18	(−)-Epicatechin gallate	75.80 ± 5.12	42.19 ± 2.13	-
19	Luteolin 7-*O*-glucuronide	-	71.35 ± 5.62	-
20	Quercetin hexoside	127.02 ± 9.56	37.03 ± 1.18	-
21	Luteolin-7-*O*-rutinoside	65.15 ± 4.36	66.52 ± 3.49	-

**Table 4:** Antioxidant activity of different maturity stages of *C. moschata* polyphenols.

Stages of Ripeness	DPPH (μmol TE g^−1^ FW)	ABTS (μmol TE g^−1^ FW)
Young	0.053 ± 0.008	0.067 ± 0.048
Mature	0.065 ± 0.010	0.074 ± 0.021
Ripened	0.048 ± 0.005	0.055 ± 0.003

**Table 5:** Antimicrobial activity of different maturity stages of *C. moschata* polyphenols.

Strains	Young (mm)	Mature (mm)	Ripened (mm)	MIC (mg L^−1^)
*E. hirae*	6 ± 0	7.16 ± 0.38	4 ± 0	2.5
*P. mirabilis*	4.16 ± 0.32	5.33 ± 0.50	3.33 ± 1.25	5
*B. subtilis*	5 ± 0	6.25 ± 0.25	3.75 ± 0.89	5
*P. aeruginosa*	4.75 ± 2.57	6 ± 0	3.5 ± 0.5	5
*S. dysenteriae*	5.66 ± 0.54	9.10 ± 1.26	5.5 ± 0.81	1.25
*S. aureus*	10.66 ± 0.60	12 ± 0	9.75 ± 0.4	0.75
*E. coli*	6 ± 0.	8.33 ± 0.87	5.25 ± 0.64	1.25
*L. monocytogenes*	5 ± 0	6.58 ± 2.02	4 ± 0	2.5
*S. typhimurium*	4.5 ± 3.39	5 ± 0	3 ± 0	5
*B. animalis* sbsp *lactis*	-	-	-	-
*Lb. rhamnosus*	-	-	-	-

## Data Availability

Not applicable.

## Sample Availability

Samples of the pumpkins are available from the authors.
